# Challenge of Liquid Stressed Protective Materials and Environmental Persistence of Ebola Virus

**DOI:** 10.1038/s41598-017-04137-2

**Published:** 2017-06-29

**Authors:** Aidan M. Nikiforuk, Todd A. Cutts, Steven S. Theriault, Bradley W. M. Cook

**Affiliations:** 1 Applied Biosafety Research Program, Canadian Science Centre for Human and Animal Health, 1015 Arlington Street, Winnipeg, MB R3E 3P6 Canada; 2 J. C. Wilt Infectious Diseases Research Centre, Public Health Agency of Canada, 745 Logan Street, Winnipeg, MB R3E 3L5 Canada; 3 Department of Microbiology, The University of Manitoba, Winnipeg, MB R3T 2N2 Canada

## Abstract

After the largest Ebola virus outbreak in history, experts have attempted to answer how the Zaire ebolavirus species emerged in West Africa and caused chains of human-to-human transmission. The widespread and untimely infection of Health Care Workers (HCW) in the affected countries accelerated spread of the virus within the community. Among the reasons attributed to this trend, it must be considered that HCW were exposed to the virus in their occupational environment. The contribution of environmental conditions to the spread of Ebola in West Africa was examined by investigating the effect of temperature/humidity on the virus’s environmental persistence and by modeling if saturation (liquid stress) allows for penetration of Ebola virus through personal protective equipment (PPE). Ebola-Makona virus persisted on PPE and materials found in outbreak settings for less than 72 hours at 27 °C and 80% relative humidity (RH). A difference in virus penetration was observed between dry (5%, 1/21 tests) and saturated (33%, 7/21 tests) samples of PPE. Infectious virus particles penetrated through saturated coupons of Tyvek Micro Clean, Tychem QC, whole surgical masks and N95 respirators. These findings suggest inclusion of saturation or similar liquid stress simulation in protective equipment testing standards.

## Introduction

The Ebola virus, a member of the *Filoviridae*, has caused more than 20 sporadic and lethal outbreaks since its discovery in central Africa during 1976^1, 2^. From December 2013 to March 2016, the virus became an international concern due to the largest known outbreak in six West African nations including Liberia, Sierra Leone and Guinea^3^.

Until recently, little has been known about how the Ebola virus behaves in the environment outside of a host organism. Experts believed the virus was relatively weak with a short half-life and much environmental sensitivity^4–6^. Contemporary studies suggest reconsideration of how the virus behaves and survives in the environment^7^. Infectious virus particles have been recovered from materials frequently used in clinical settings up to 172 hours^8^ and in outbreak conditions from 72–240 hours^7, 9^. Epidemiological reports have cited a risk of acquiring Ebola from environmental sources such as the bodies of the deceased, excrement and contaminated medical equipment^10^. Climate also appears to heavily influence Ebola virus emergence, as most Ebola epidemics start with the end of the rainy season in December and January when temperature and humidity are both high^11^. On the front lines of Ebola outbreaks are HCW who volunteer to care for those sick with Ebola virus disease, a disturbingly high incidence of HCW infections was observed throughout the West African Ebola outbreak (3.9%, 815/20,955 of total cases) which was highest during the record-setting temperatures of early 2014^11, 12^. The average rate of HCW infections peaked to 12.3% of the total during January, February and March, a greater incidence than any other three consecutive month period of the year^13^. The percentage of HCW infections per total infections decreased over the course of the outbreak suggesting the effectiveness of Ebola virus containment efforts including barrier protection, use of PPE, sanitary burial, epidemiological tracking and field diagnostics^3^.

Besides dealing with high temperatures and humidity, aid workers combatting the Ebola outbreak in West Africa were challenged by the occupational risk of fluid-borne virus exposure (i.e. intubation), and having to routinely wear/remove standard Ebola virus PPE^14–16^. In Ebola treatment units (ETU), personnel wear: rubber boots, a full length Tychem QC suit, a Tyvek Micro-Clean hood, googles, facial mask or respirator and two pairs of nitrile gloves^17, 18^.The burden of this PPE, low gaseous permeability of the materials and, increased body temperature due to insulation and the retention of heat limit the shift length of HCW from half to a full hour^19^. Even short shifts do not prevent excessive sweating and heat exhaustion^17^. Perhaps temperature and humidity contributed to the high incidence of HCW infections during early 2014 as PPE may have become saturated by: perspiration, humidified air, condensation during respiration or bodily fluid exposure during use^20^. Saturation-thorough wetting- has been identified by the American Society for Testing and Materials (ASTM) as a stressor of protective materials capable of impacting the integrity of their protective barrier and performance^21^. Ebola virus may penetrate through liquid stressed material increasing the risk of infection.

We performed three experiments (Environmental Persistence, PPE Penetration and Mask Penetration) to investigate how the Ebola-Makona virus persists outside a host in West African climatic conditions and if saturation with phosphate-buffered saline (PBS) affects the permeability of protective materials (i.e. PPE) worn by HCW responding to an outbreak.

## Results

### Environmental Persistence

**Figure 1:**
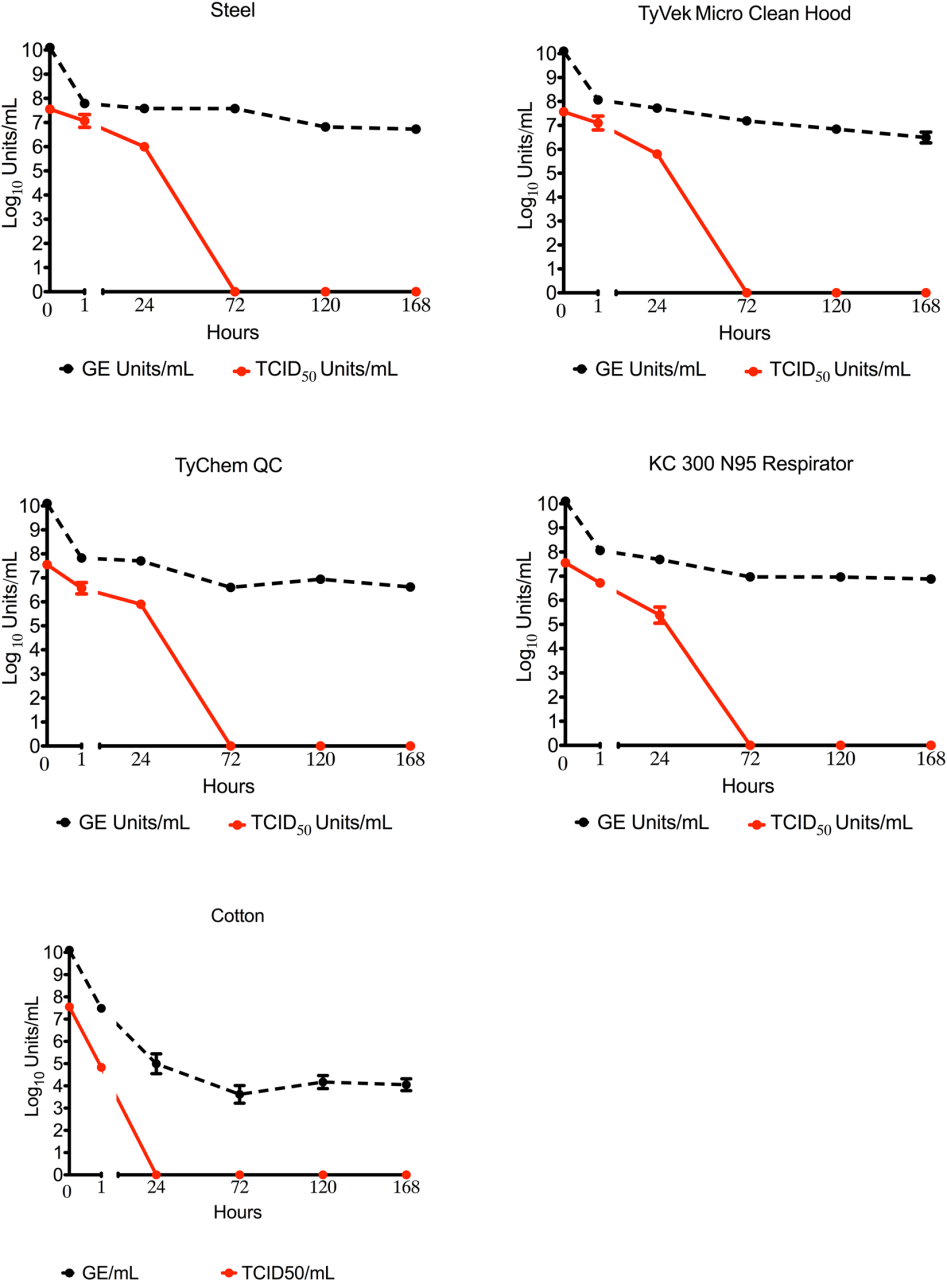
Persistence of infectious Ebola virus and viral RNA on PPE and ETU materials. Ebola virus was recovered from materials incubated in average West African climate conditions (27 °C and 80% RH) using a variation of the QCT-2 protocol published as ASTM E2197 – 11. The Environmental Persistence experiments were performed in three biological replicates, each consisting of three technical repetitions. Reported values are the average across all replicates (n = 9) error was calculated as standard error of the mean. Where: GE- genome equivalents and TCID_50_- tissue culture infectious dose fifty.

The ability for Ebola virus to remain viable on surfaces in average West African climatic conditions 27 °C and 80% RH has implications for fomite-driven transmission and the effectiveness of barrier protection. Ebola-Makona remained viable on all the tested materials between 24 to 72 hours post-inoculation with the exception of gloves (<1 hour) and, cotton and goggles (<24 hours). No virus was detected at 72-hours on any of materials (Figs 1 and S1). Ebola virus viral RNA (vRNA) was not as significantly impacted, the quantities of vRNA recovered from each material remained consistent throughout the experiment (genome equivalents/mL ranged from 4.1 (+/−0.3) to 7.1 (+/−0.1) with respective Ct values of 27.8 (+/−0.9) to 17.4 (+/−0.4) at 168 hours post-inoculation), despite the absence of infectious virus particles (Table S1). The continued detection of vRNA indicates that it does not strongly correlate with infectious virus particles in the environment.

### PPE Penetration

**Figure 2:**
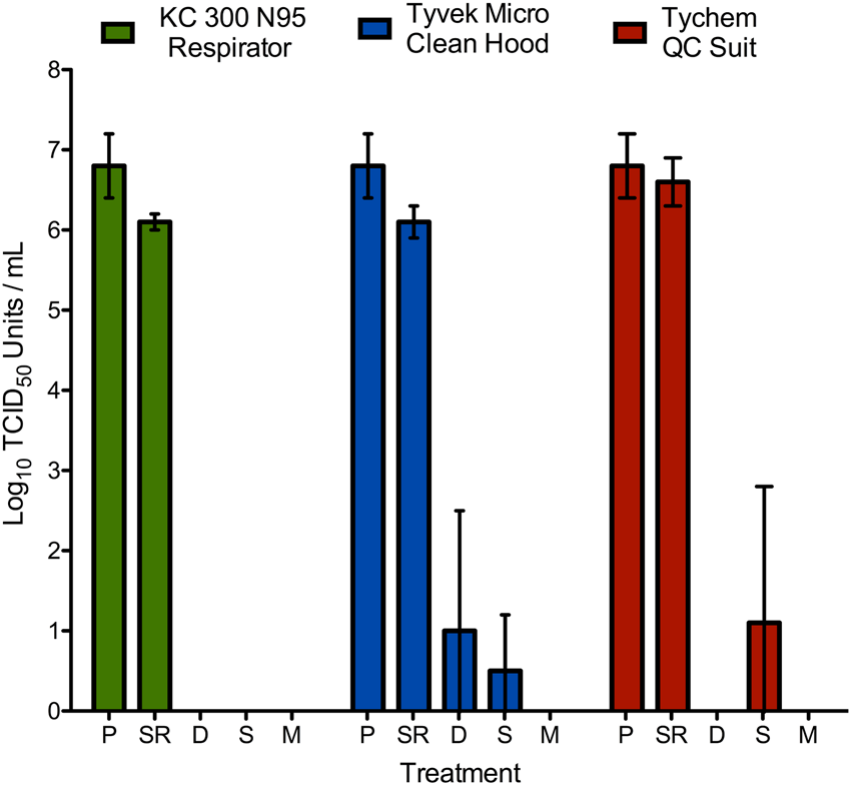
Ebola virus penetration through three types of protective material, which allowed the virus to persist for ≥24 hrs at 27 °C and 80% relative humidity. Virus was inoculated onto the surface of a dry or saturated coupon and left to incubate at 21.5 °C and 30–35% RH for 30 minutes. The coupons were then swabbed on the exposed and unexposed side to recover the virus used in the challenge and detect viral penetration through the material. Where: P- positive (inoculation/challenge) control, SR- swab recovery, D- dry sample, S-saturated sample, M- mock. The PPE Penetration experiments were performed in four biological replicates, each consisting of one technical repetition. Reported values are the average across all replicates (n = 4) error was calculated as standard error of the mean.

The hot and humid conditions of West Africa cause staff working in ETU or other outbreak facilities to suffer from elevated body temperatures leading to heavy perspiration, heat exhaustion and increased respiration rate. The saturation of PPE in such conditions may allow for virus particles which persist on a material to penetrate through causing a risk of exposure. To elucidate a link between virus penetration and vulnerability of saturated material, we tested for passage of Ebola virus through coupons of three PPE materials, which showed longer term virus survivability in the persistence study. Dry and saturated coupons made from a Tyvek Micro-Clean hood, Tychem QC suit and KC 300 N95 respirator (including fluid-shield) were inoculated with 6.8 (+/−0.4) log_10_ TCID_50_/mL of Ebola-Makona virus. Dry coupons made from Tychem QC did not allow for viral penetration. Ebola did penetrate through a dry coupon made from the Tyvek Micro-Clean hood, 3.8 log_10_ TCID_50_ units/mL was recovered in 1/4 replicates. Coupons soaked in PBS, meant to simulate saturation, provided lesser protection than dry material, as both the Tyvek hood and the Tychem QC suit had a failure rate of 1/4. In the instances of failure, 1.8 log_10_ TCID_50_ units/mL of Ebola virus penetrated the Tyvek Micro-Clean hood and 4.6 log_10_ TCID_50_ units/mL passed through Tychem QC suit. These titres were averaged across the four replicates to 0.5 (+/−0.7) log_10_ TCID_50_/mL for the hood and 1.1 (+/−1.7) log_10_ TCID_50_ units/mL for the suit (Fig. 2). Virus was not recovered from the dry or saturated KC 300 N95 mask coupons (Fig. 2).

### Mask Penetration

**Figure 3:**
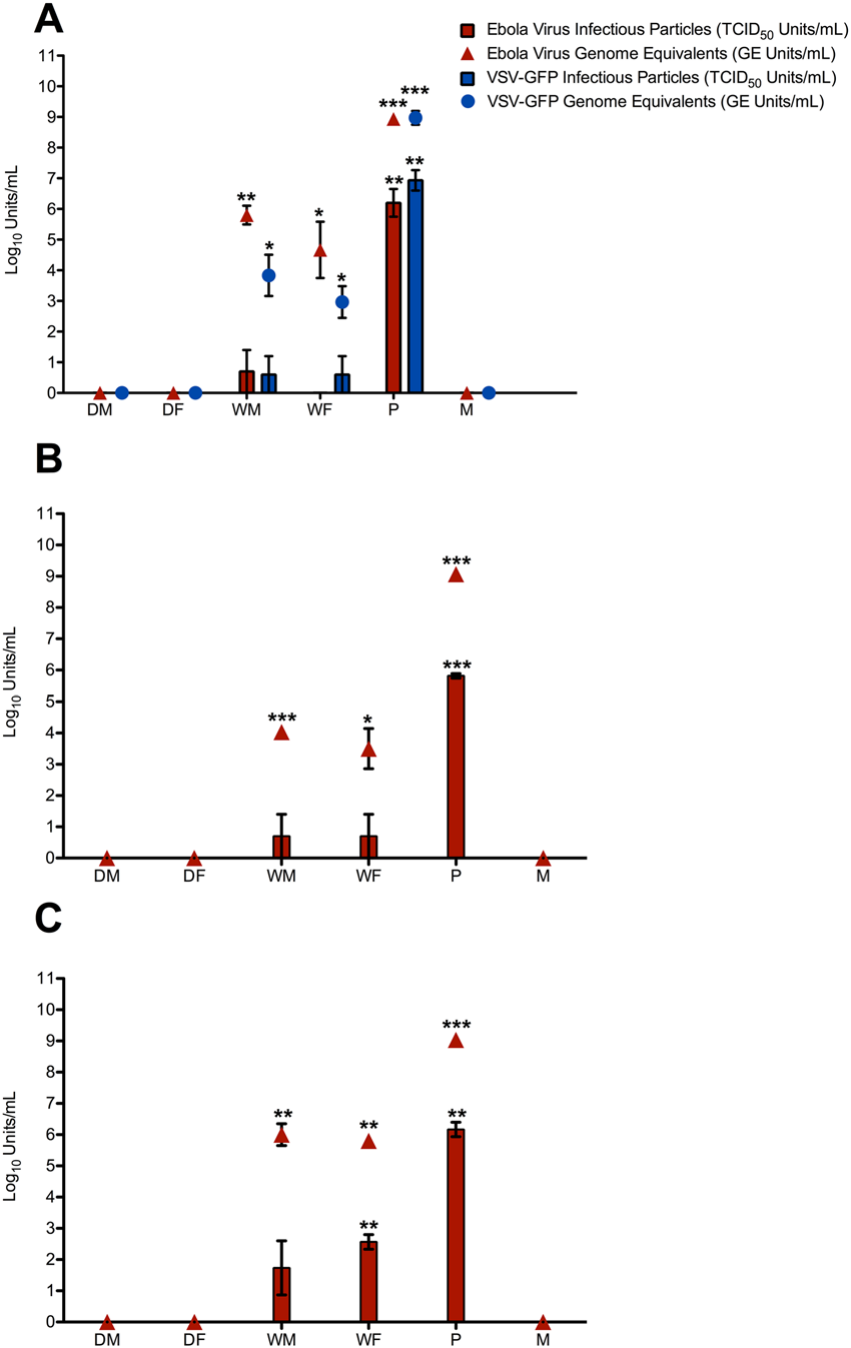
Ebola or VSV-GFP virus penetration through a surgical mask and N95 respirators challenged in dry and saturated conditions. Whole masks were fitted onto the face of a respiratory mannequin and challenged with Ebola virus. The mannequin was programmed to breath for thirty minutes at 21.5 °C and 30–35% RH. The masks were then removed and the inside surface of the mask and face of the mannequin were dry swabbed to detect virus penetration: (**A**) surgical mask, (**B**) KC 200 N95 respirator, (**C**) KC 300 N95 respirator. An equal volume of virus used to challenge the masks was back titrated as a positive control. Recovery of vRNA but not infectious virus particles suggest that saturation of the examined masks compromised protection by 100%. Where: DM-dry mask, DF- dry face, WM- wet mask, WF- wet face, P-positive/inoculation control, M-mock. The Mask Penetration experiments were performed in three biological replicates, each consisting of three technical repetitions. Reported values are the average across all replicates (n = 9) error was calculated as standard error of the mean. A paired T-test was used to calculate significant differences in mean values between the test and negative control (P-value: not significant >0.05, *≤0.05, **≤0.01 and ***≤0.001).

Ebola virus penetrated through saturated coupons of Tyvek Micro-Clean and Tychem QC, but not through the KC 300-N95 mask. We wished to further investigate if Ebola virus could penetrate surgical masks or N95 respirators. The KC 300 mask coupons used in the persistence study were not representative of the entire mask surface, because they were made from the area surrounding the facial opening which is lined with a plastic fluid shield. The fluid shield layer may have prevented detection of virus that had penetrated through the other layers of the mask. To account for the bias of testing coupons made from a part of the whole material, we challenged whole masks with Ebola virus. Three types of facial protection (surgical mask, KC 200-N95 respirator and KC 300-N95 respirator) were challenged with virus using a respiratory mannequin. Saturating the masks in PBS, affected the equipment’s permeability to Ebola virus particles. Regardless of mask type, saturation allowed for virus penetration in at least 1/3 biological replicates of the experiment. Virus particles were also detected on the face of the mannequin after removal of saturated KC 200 and KC 300 N95 respirators (Fig. 3B,C). In comparison, no virus was detected from the inside surface of any dry masks or from the mannequin’s face when fitted with a dry mask (Fig. 3A–C). RNA was recovered from all of the saturated mask samples and positive controls, but not from the dry mask samples. In cases where both infectious virus particles and vRNA were recovered, the number of genomic equivalents was ~1–4 log_10_ units/mL higher than the titre of infectious virus particles (Fig. 3A–C). The recovery of vRNA from mask and face swabs taken from all the saturated mask samples (9/9) indicates that each mask was likely penetrated by infectious virus particles (Table S3). In cases where vRNA was detected in the absence of infectious virus particles, the amount of virus was below the detection limit for the TCID_50_ assay.

The surgical mask was also challenged with Vesicular stomatitis virus engineered to express green fluorescent protein (VSV-GFP), a potential surrogate for Zaire ebolavirus using the same conditions (Fig. 3A). Infectious VSV-GFP was not recovered from any of the dry masks or the face of the mannequin when fitted with a dry mask (0/3 replicates). Infectious virus particles were detected from the inside surface of the saturated mask and face of the mannequin in 1/3 experiments (Fig. 3A). Viral RNA was only detected in saturated mask samples and in the controls, no vRNA was detected from swabbing the dry masks or the mannequin when fitted with a dry mask. In the samples where both infectious virus particles and vRNA was recovered, the number of genomic equivalents was ~1–3 log_10_ units/mL higher than the titer of infectious virus particles (Table S4). vRNA was recovered from swabbing the inner surface of the saturated masks and face of the mannequin fitted with saturated masks in 3/3 replicates. This result indicates that infectious virus particles likely penetrated each saturated surgical mask; however, the amounts were too low for detection by TCID_50_ assay.

## Discussion

Virologists understanding of the Ebola virus has evolved since the end of the Makona outbreak because its severity and scale raised difficult questions and challenges^22–24^. The ramifications of the outbreak cultivated our study and the findings suggest reconsideration of how to best protect HCW and others with the risk of Ebola virus exposure in outbreak environments. High temperature and humidity were demonstrated to shorten Ebola virus persistence on the challenged materials in comparison to conditions reflective of a clinical environment in resource wealthy nations^7^. Notably, virus persisted long enough (>24 hours) to allow for possible fomite driven transmission if contaminated surfaces are not properly identified and disinfected^25^. Other authors found that material and fluid matrix types complicate defining how temperature and humidity affect Ebola virus persistence. At high temperature and humidity, the virus survives longest in whole human blood^7, 9^. The survivability of Ebola in whole blood - being longer than that of residual bodily fluids - could lengthen the window of opportunity for the virus to penetrate through material. Liquid stress/saturation of PPE, a consequence of hot humid conditions, perspiration and condensation from respiration, compromised the protection of: spun polyester attire, whole surgical masks and N95 respirators. Ebola virus penetrated through saturated materials at a greater incidence (n = 7/21) than the same materials when dry (n = 1/21)(Tables S2 and S3). The penetration of Ebola virus through a dry coupon made from a Dupont Tyvek Micro-Clean hood is not unexpected as the ASTM F2101 standard rated the equipment’s 3.0 µm filtration efficiency as 98.9% +/− 1.2% STD^26^. Persons responsible for the selection of PPE during Ebola virus outbreaks should check filtration efficiency ratings and other descriptors provided by regulators before approval. The average titre of infectious virus particles recovered from the seven samples (1.1 log_10_ TCID_50_ units/mL) is greater than the perceived infectious dose^27^. In agreement with our previous work, recovery of Ebola virus RNA does not adequately represent infectious virus particles in stressful environmental conditions (i.e. chemical disinfection or desiccation)^25^. Detection of vRNA in the mask penetration studies was more comparable to the titre of infectious virus particles, when they were detected together. Current standards for assessing PPE do not include saturation (i.e. perspiration) as a factor in their testing models. The passage of Ebola virus and the tested surrogate virus VSV-GFP through the examined materials should not be completely unexpected because, many items of PPE are certified to protect against penetration of particles ≥0.1–0.3 µm in size^28, 29^. Ebola virus particles measure 80 nm (0.080 µm) in diameter and vary in length up to 14,000 nm (14 µm)^30^. VSV was chosen as an Ebola virus surrogate because it also possesses an envelope and measures similarly (70 nm (0.070 µm) in diameter)^31^. Both the Ebola and VSV particle sizes fall below the defined minimum pore size for each of the tested masks and the woven polyester fabric used to manufacture Tyvek and Tychem QC materials (Table S5). Viral penetration through the material pores was likely increased after saturation because of changes in pore structure, surface tension, fluid polarity and capillary action^32^.

The conditions applied in this study surpassed the recommendations for PPE use provided by the manufacturers and applied in current standards. Kimberly Clark recommends that neither the KC 200 or KC 300 N95 respirators be used in elevated temperature (>25 °C) or relative humidity (>60%). Dupont does not recommend the use of the Tyvek Micro Clean hood for biohazard protection but for use in controlled environments such as clean-rooms. The ASTM standards, condition fabric samples for a minimum of 24 hours to 21 +/− 5 °C and 30–80% relative humidity^32^. Other limitations of our study include not measuring the surface tension of liquids on the material coupons or measuring the volume of PBS each mask absorbed after 30 minutes. We acknowledge that the use of PBS (pH 7) may not accurately represent perspiration of a human being, one of the pre-defined causes of saturation. However, it would be difficult to more closely model how human sweat directly affects the permeability of protective materials, because of the complex interactions between the solute, material and other environmental factors.

The possible penetration of Ebola virus through liquid stressed (saturated) PPE should impact future concepts and strategies of infection prevention and control in tropical environments. The negative impacts of saturation on the protection afforded by PPE should be considered and mitigated. PPE could either be re-designed for increased temperature transfer/gaseous exchange to reduce perspiration or to offer more complete protection^19^. The use of powered air purifying respirators should be discussed as they provide air circulation and liquid impermeable shielding to the wearer’s face. The face is considered the most vulnerable region of the body to Ebola virus infection due to mucosal membranes of the eyes, nose and mouth^30^. Even the use of highly protective PPE does not guarantee safety of HCWs or others exposed to Ebola virus because user error (i.e. improper donning/doffing or use of the equipment) may lead to exposure or heighten exposure risk^33^.

Continued investigation into the high incidence of HCW infections during the Ebola-Makona outbreak in West Africa has attributed numerous factors including lack of suitable PPE and its effective use, virus exposure in the community, reporting bias in epidemiological numbers and propensity for colleagues to treat each other forgoing personal safety^3, 13^. The contributions of the West African environment also deserve consideration. Teams deployed to the Ebola crisis in Guinea reported that excessive deforestation around ETU made daily temperatures very hot^34^. Such extreme environmental conditions paired with the physical exertion of work within ETU likely caused HCW to perspire and the build-up of condensation on the inner surface of their equipment. These sources of saturation may have increased the risk of Ebola virus exposure. Although our understanding of Ebola is evolving, many questions remain. Specifically, can the PPE used in Ebola outbreaks be made more safe? And do current testing standards accommodate for the stress of outbreak scenarios?

We propose a new model for testing the protection that Ebola virus PPE affords to the wearer which includes saturation (liquid stressing) of the tested materials in PBS or a like simulant of perspiration or condensation. Spun polyester fabrics used to manufacture PPE such as Tychem, Tyvek are subjected to the ISO 16604, ASTM F1670 and ASTM F1671 as benchmarks of liquid permeability and virus penetration^21, 32, 35, 36^. Masks are evaluated by three ASTM standards (F1862, F2101 and F2299) which test penetration of horizontal projection of synthetic blood, bacterial filtration efficiency and particulate penetration using latex spheres^37–39^. None of these standard tests factor for saturation/perspiration or challenge the material with infectious Ebola virus. In fact, the ASTM reference document F1671 states that protection afforded by protective material must be assessed on a pathogen-specific basis^21^. Reviews on the usage of PPE in Ebola virus outbreaks call for more empirical evidence to aid in the selection of appropriate materials^14, 40^. As a solution they suggest that items of PPE should undergo testing in a BSL-4 facility following ISO or ASTM methodology and challenged with Ebola instead of a surrogate virus^17^. The bacteriophage Phi-X174 functions as a surrogate for blood-borne viruses in the ISO 16604 standard. We were unable to perform the standard using Ebola virus, because it requires a positive pressure apparatus not currently approved for use in high containment laboratories. Future efforts should aim to complete the aforementioned standards including saturation as a preconditioning factor with the Ebola virus or VSV as a surrogate. The methods of this work follow an amended QCT-2 method published as ASTM E2197 – 11 and by the US Environmental Protection Agency^41, 42^. Generating knowledge of Ebola in environmental systems particularly the West African climate, will allow for better policy on hemorrhagic fever virus infection prevention control and outbreak management.

### Limitations of the Study

We wish to highlight that real world use of PPE may involve complex potentially risk amplifying conditions not recommended by the manufacturer or tested in standards. The experimental model we devised- liquid stress of PPE and challenge with high titre infectious Ebola virus- was intended to demonstrate a worst case scenario. This scenario did not incorporate available strategies to decrease the examined risks such as disinfection and removal of affected equipment. We hope that this work helps policy makers define acceptable levels of liquid stress such as perspiration or condensation during use of PPE.

## Materials and Methods

### Virus Stock Preparations

Ebola virus Makona variant stocks (Ebola virus/H.sapiens-tc/GIN/2014/Makona-C05) were propagated in African green monkey kidney (VeroE6) cells (ATCC CRL-1586) within the Containment Level 4 (CL-4) laboratory at the National Microbiology Laboratory (NML) of the Canadian Science Centre for Human and Animal Health (CSCHAH) located in Winnipeg, Manitoba, Canada. When approximately 80–90% CPE was observed (10–12 days post-infection), virus was harvested from supernatants by centrifugation: 5000 × g for 10 min and 108,000 × g for 2 hr with a 20% sucrose cushion. Stocks were prepared by suspending pelleted virus from the first centrifugation step (un-concentrated stock) or second centrifugation step (concentrated stock) in in Dulbecco’s modified eagle medium (DMEM)(HyClone) containing 2% Fetal Bovine Serum (FBS)(Gibco) and 1% antibiotics (Penicillin-Streptomycin) (Gibco) and stored at −80 °C. The concentrated stocks were used for the environmental persistence and PPE penetration experiments; the non-concentrated stocks were used for the mask protection experiment.

### Environmental Persistence

Carriers measuring 1.5 inches in diameter were prepared from PPE and environmental materials, they were gamma-irradiated (1 MRad) and placed inside 12 well tissue culture plates (Corning) prior to inoculation. The PPE materials included: Tyvek Micro-Clean hood and mask (Dupont) the mask was removed, Tychem QC suit (Dupont), Sol-Knit 39–124 green nitrile-coated gloves (Ansell), Flex-seal goggles (Uvex), KC 300 Fluidshield N95 particulate filter respirator including the fluid-shield layer (Kimberley-Clark) and Hazmax Kneeboot boots (Onguard). The environmental materials included: cotton-polyester pillow case, maple wood with an epoxy (CSCHAH), plastic and stainless steel (CSCHAH).

The virus inoculum was designed to mimic virus contained within residual bodily fluids from an infected Ebola patient (summarized previously Cook *et al*. 2015^7^) to accomplish this an organic soil load composed of 12.5 μL 5% bovine serum albumin (Sigma), 17.5 μL 5% tryptone (Becton Dickinson) and 50 μL 0.4% bovine mucin (Sigma) was mixed with 125 µl of Ebola-Makona. Using a positive displacement pipette, 10 µl of the virus-soil load mixture was added to each material in triplicate and an additional 10 µl added to 990 µl DMEM (2%FBS/1% antibiotics) as a positive control. The carriers were incubated at 27 °C with 80% relative humidity supplied by an electronic humidifier (Cigar Oasis) and measured daily using a digital hygrometer (Xikar). At the desired collection time point, virus was eluted from each carrier type by repeated scraping and washing with 990 µl DMEM (2% FBS/1% antibiotics) and stored at −80 °C for processing for TCID_50_ and qRT-PCR quantification.

### PPE Penetration

Carriers 2–3 inches in diameter were prepared from Tyvek Micro-Clean hood (Dupont) with the mask removed, Tychem QC coveralls (Dupont) and KC 300 Fluidshield N95 particulate filter respirator including the fluid-shield (Kimberley-Clark). The carriers were gamma-irradiated (1 MRad) and placed inside of 6 well tissue culture plates (Corning). The saturated samples were soaked in sterile PBS (pH 7) for 30–45 minutes prior to testing. Ten µl of the soil load-virus mixture was added with a positive displacement pipette onto each PPE type, in quadruplicate, independent experiments. As an positive (inoculation) control, 10 µl of Ebola-Makona was added to 990 µl of DMEM (2% FBS/1% antibiotics). After 30 minutes of incubation, each sample-type (swab recovery control, dry and saturated) was grouped and processed on the lid of a 6 well tissue culture plate. Using sterile forceps all the samples were moved to clean 6-well plates and swabbed on either the inoculated surface-as a control (recovery control)- or the unexposed side. Swabbing was performed with dry rectangular foam swabs (Texwipe, STX712A), both sides of the swabs were used to maximize virus recovery. Virus was eluted from each swab in 990 µl of DMEM (2% FBS/1% antibiotics) within a 14 mL Falcon tube (Corning). The tips of the swabs were expunged on the inside of the tube three times before removal. The swab supernatants were processed for TCID_50_ assay (500 µl) and qRT-PCR (140 µl).

### Mask Protection

Three types of facial protection were challenged with Ebola-Makona virus in dry and saturated conditions. A flat surgical mask (Harmony Ear Loop Face Mask, Adenna) and two duck-bill N95 respirators KC 200 and KC 300 (Kimberly Clark) were included in the study, the KC 300 respirator was actively used during the Makona outbreak, the other two masks or similar designs may have been available in resource limited areas. Six masks of each type were packaged and gamma-irradiated at 1 MRad, before use in CL-4. Dry masks were fitted directly onto the face of a mannequin (Dry Active Respiratory Thermal Head, Measurement Technology Northwest) capable of simulating human respiratory action. Saturated masks were placed in 1 L of sterile PBS and left to absorb liquid for 30 minutes. Four 200 μl inoculations of Ebola-Makona stock were added to the outside surface of the dry or saturated masks in the four quadrants of a cross centering on the nose. The same volume of virus 4 × 200 μl was titrated to serve as a positive (inoculation) control. The mannequin was programmed to breath at a rate of 20 breaths per minute for 30 minutes. Each inhale/exhale moved 0.5 L of air, approximately the average tidal volume of human adult lungs. At the end of the breathing cycle, the masks were removed from the face of the mannequin. The inside surface of the mask and face of the mannequin were dry swabbed with sterile foam swabs (Texwipe, STX712A). The swabs were treated identically to those used in the PPE penetration experiments. The experiment was conducted in three biological replicates for each item of facial protection in the dry and saturated test conditions.

Dry and saturated surgical masks were also challenged in CL-2 using Vesicular stomatitis virus genetically modified to express green fluorescent protein as an Ebola virus surrogate. VeroE6 Cell tissue culture positive for VSV-infection were visualized using a confocal microscope (Zeiss Axiovert 40 CFL fitted with a HB0 50 light source).

### Fifty Percent Tissue Culture Infectious Dose (TCID_50_) Assay

Virus supernatants from the environmental persistence, PPE penetration and mask protection experiments were 10-fold serially diluted and 50 µl of each dilution in triplicate was used to infect 80% confluent VeroE6 monolayers in 96-well culture plates for 1 hour at 37 °C/5% CO_2_. After virus absorption, 150 µl of DMEM (2% FBS/1% antibiotics) was added and the cells were incubated at 37 °C/5% CO_2_. Monolayers were scored for CPE on 7 and 14 days post-infection for Ebola and 1 and 2 days for VSV-GFP, titres reported as Log 10 TCID_50_ Units/mL were determined using the Reed and Muench formula^8^.

### Quantitative reverse transcription-polymerase chain reaction (qRT-PCR)

RNA was extracted from viral supernatant using commercial reagents (Viral RNA Isolation Kit, Qiagen) and quantified by qRT-PCR (Lightcycler 480 RNA Master Hydrolysis Probes, Roche). Ebola virus RNA was detected by use of a 2:1 primer probe mix (300 nm, ZEBOV LF- CAGCCAGCAATTTCTTCCAT, ZEBOV LR-TTTCGGTTGCTGTTTCTGTG: 150 nm, ZEBOV LP1 -6FAM’-ATCATTGGCGTACTGGAGGAGCAG’MGBNFQ, ZEBOV LP2- 6FAM’-TCATTGGCGTACTGGAGGAGCAGG’MGBNFQ) specific for *Zaire ebolavirus species*. The conditions of the PCR reaction were programmed to: 61 °C for 3 min, 95 °C for 30 sec, 40 cycles of 95 °C for 15 sec and 60 °C for 40 sec. VSV-GFP RNA was detected by using a primer probe set targeting the nucleoprotein region (VSV-GFP NPF- CGGAGGGACGACTAATGC, VSV-GFP NPR-CGAGCGATTCGACCACATC, probe-6FAM’CGCCACAAGGCAG’MGBNFQ). The same thermo-cycling conditions were used.

### Quantitative Analysis

Genomic equivalents per millilitre were calculated using the formula published by Chiu *et al*. (C = Q × V_DNA_/V_PCR_× 1/V_EX_× 1000)^43^. A paired T-test was used to analyze the results of the mask protection experiment in Fig. 3A–C and determine significant differences (*) between the dry to saturated samples and positive to negative controls. Significant results of the T-tests are represented as P-values: not significant >0.05, *≤0.05, **≤0.01 and ***≤0.001). All figures show average values from experimental replicates, error was calculated as standard error of the mean using GraphPad Prism.

## Electronic supplementary material

Supplementary Information
