# Supplementary material for: Challenge of Liquid Stressed Protective Materials and Environmental Persistence of Ebola Virus
Source: Sci Rep. 2017 Jun 29;7:4388. doi: 10.1038/s41598-017-04137-2 (PMC5491502; doi:10.1038/s41598-017-04137-2)
Supplement: Supplementary file 1 — Supplementary Information [file 41598_2017_4137_MOESM1_ESM.pdf]

Long Title: Challenge of Liquid Stressed Protective Materials and Environmental Persistence of Ebola Virus

Short Title: Environmental Models of Ebola Virus Transmission

Authors: Aidan M. Nikiforuk[1,2], Todd A. Cutts[1], Steven S. Theriault[1,2*], Bradley W.M. Cook[1,2]

Affiliations:

[1]Applied Biosafety Research Program, Canadian Science Centre for Human and Animal Health and J. C. Wilt Infectious Diseases Research Centre, Public Health Agency of Canada, 1015 Arlington Street, Winnipeg, MB R3E 3P6, Canada and 745 Logan Street, Winnipeg, MB R3E 3L5, Canada.
[2]Department of Microbiology, The University of Manitoba, Winnipeg, MB R3T 2N2, Canada.

*Dr. Steven Theriault, MSc., Ph.D., Applied Biosafety Research Program, Public Health Agency of Canada, 1015 Arlington Street, Winnipeg, MB, Canada (steven.theriault@phac-aspc.gc.ca).

Supplementary Information:

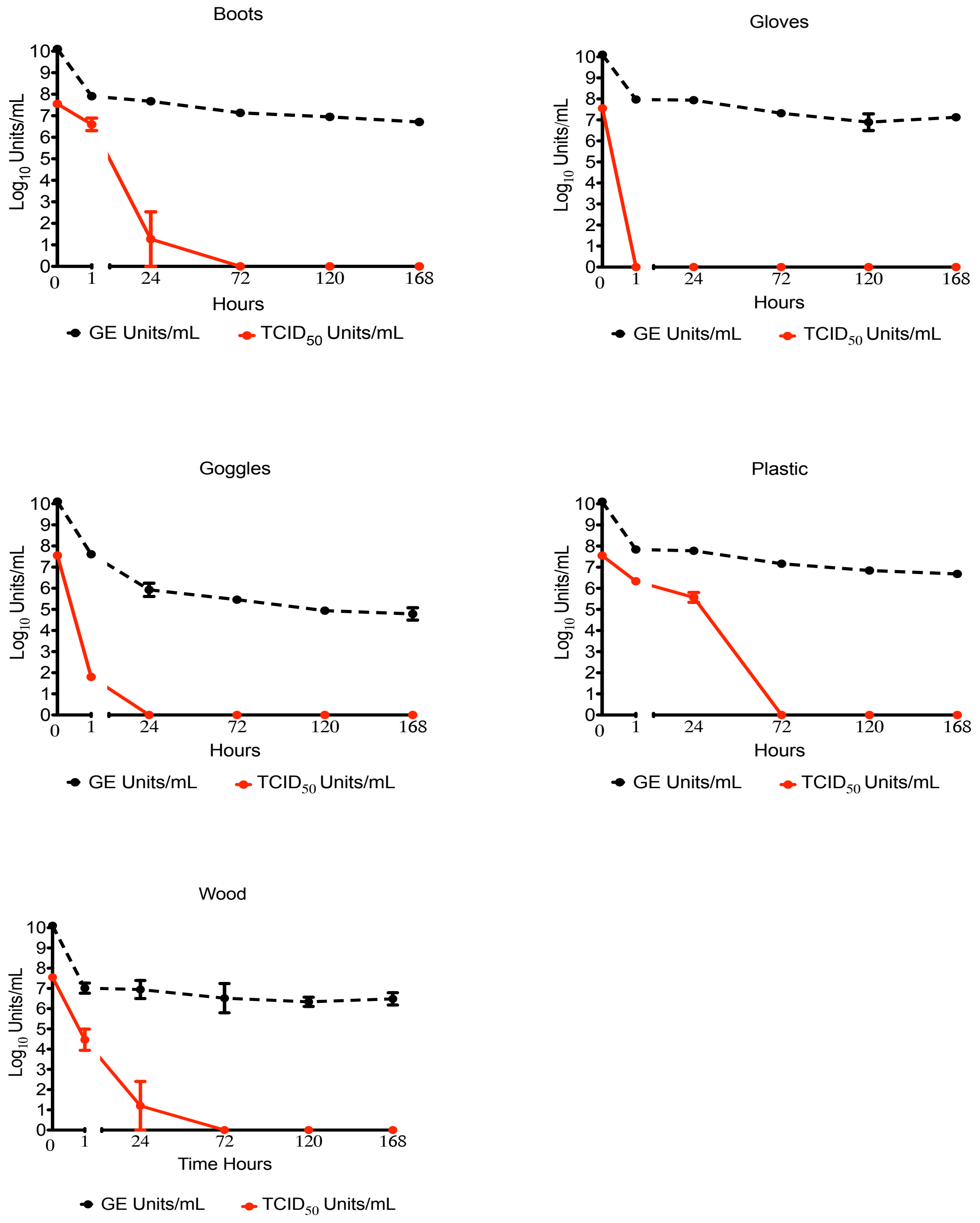

Fig S1. Persistence of infectious Ebola virus and viral RNA on PPE and ETU material. Ebola virus was recovered from materials incubated at West African climate conditions using a variation of the QCT-2 protocol published as ASTM E2197 – 11. The Environmental Persistence experiments were performed in three biological replicates, each consisting of three technical repetitions. Reported values are the average across all replicates (n=9) error was calculated as standard error of the mean. Where: GE- genome equivalents and $TCID_{50}$- tissue culture infectious dose fifty.

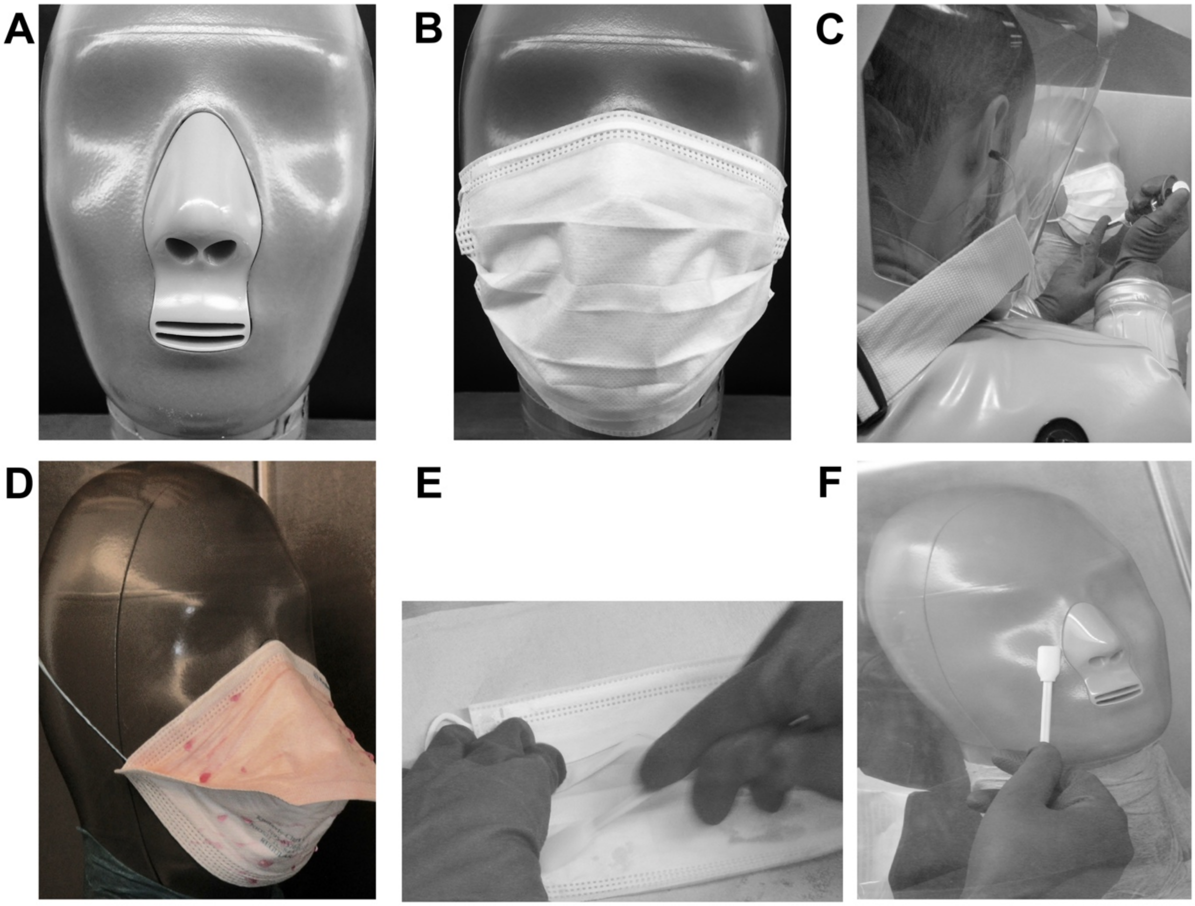

Fig S2. Images captured from CL-4 showing steps in the process of the Mask Penetration experiment. A) the mannequin head used in the mask penetration experiments. B) The mannequin head fitted with the surgical mask. C) Challenging a mask fitted onto the mannequin head with Ebola virus. D) Virus on the outside surface of a challenged mask. E) Swabbing the inside surface of a tested mask. F) Swabbing the face of the mannequin after removing a challenged mask.

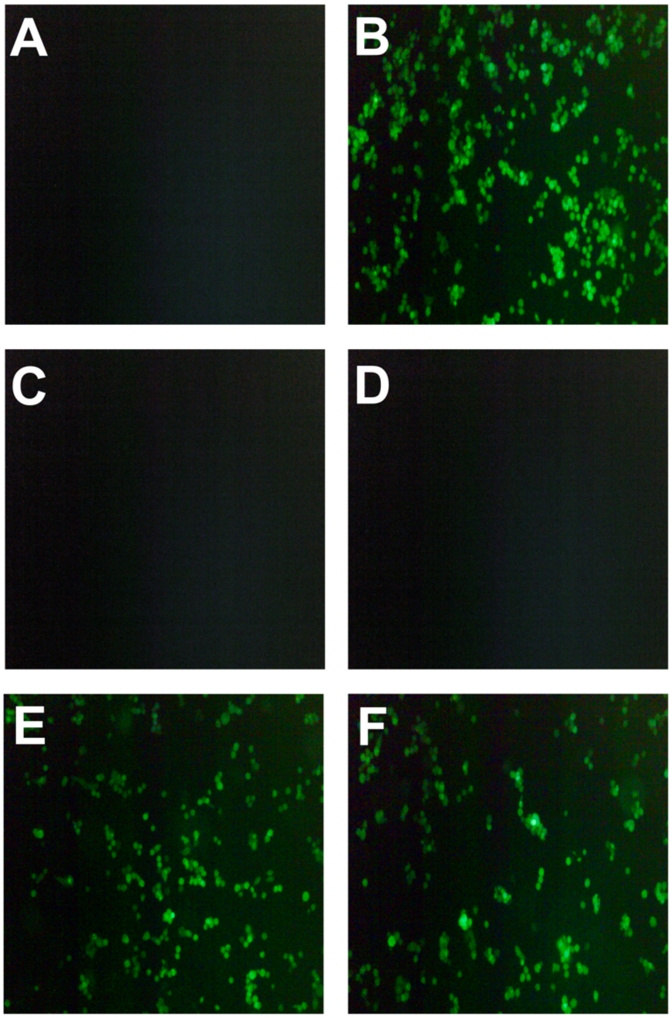

Fig S3. Penetration of VSV-GFP through a surgical mask fitted to the face of the respiratory mannequin. Infectious virus was viewed using fluorescent microscopy (4x), 24 post infection, to detect the expression of GFP from the viral genome: A) negative control, B) positive control, C) dry mask, mask swab, D) dry mask, face swab, E) saturated mask, mask swab, F) saturated mask, face swab.

Table S1: Ebola virus persistence on select items of PPE from 0 to 168 hours at 27°C and 80% RH measured in infectious virus titre, cycle threshold and genomic equivalents.

Material	Time (Hours)	VT *	Ct †	GE ‡
**Inoculation Control**	0	7.6 (+/- 0.2)	12.6 (+/-0.3)	10.2 (+/-0.1)
**Wood**	1	4.4 (+/- 0.5)	19.1 (+/- 0.8)	7.0 (+/- 0.3)
**Plastic**	1	6.3 (+/- 0.2)	16.6 (+/- 0.1)	7.8 (+/- 0.0)
**Steel**	1	7.1 (+/- 0.4)	16.7 (+/- 0.6)	7.8 (+/- 0.2)
**Cotton**	1	4.8 (+/- 0.2)	17.6 (+/- 0.4)	7.5 (+/- 0.1)
**Tyvek Micro -Clean Hood**	1	7.1 (+/- 0.3)	15.9 (+/- 0.1)	8.1 (+/- 0.0)
**Tychem QC Suit**	1	6.6 (+/- 0.4)	16.6 (+/- 0.2)	7.8 (+/- 0.1)
**Goggles**	1	1.8 (+/- 0.0)	17.3 (+/- 0.5)	7.6 (+/- 0.2)
**N95 Mask**	1	6.7 (+/- 0.1)	15.9 (+/- 0.1)	8.1 (+/- 0.0)
**Gloves**	1	0.0 (+/- 0.0)	16.1 (+/- 0.2)	8.0 (+/- 0.1)
**Boots**	1	6.6 (+/- 0.3)	16.3 (+/- 0.2)	7.9 (+/- 0.1)
**Wood**	24	1.2 (+/- 1.6)	19.3 (+/- 1.4)	6.9 (+/- 0.4)
**Plastic**	24	5.6 (+/- 0.4)	16.7 (+/- 0.4)	7.8 (+/- 0.1)
**Steel**	24	6.0 (+/- 0.1)	17.4 (+/- 0.2)	7.6 (+/- 0.1)
**Cotton**	24	0.0 (+/- 0.0)	25.3 (+/- 1.4)	5.0 (+/- 0.4)
**Tyvek Micro- Clean Hood**	24	5.8 (+/- 0.0)	16.9 (+/- 0.1)	7.7 (+/- 0.0)
**Tychem QC Suit**	24	5.9 (+/- 0.1)	17.0 (+/- 0.4)	7.7 (+/- 0.1)
**Goggles**	24	0.0 (+/- 0.0)	22.5 (+/- 1.0)	5.9 (+/- 0.3)

**Kimberly-Clark 300 N95 Respirator**	24	5.4 (+/- 0.4)	17.0 (+/- 0.1)	7.7 (+/- 0.0)
**Gloves**	24	0.0 (+/- 0.0)	16.2 (+/- 0.1)	7.9 (+/- 0.0)
**Boots**	24	1.3 (+/- 1.7)	17.1 (+/- 0.2)	7.7 (+/- 0.1)
**Wood**	72	0.0 (+/- 0.0)	20.6 (+/- 2.2)	6.5 (+/- 0.7)
**Plastic**	72	0.0 (+/- 0.0)	18.6 (+/- 0.3)	7.2 (+/- 0.1)
**Steel**	72	0.0 (+/- 0.0)	17.4 (+/- 0.1)	7.6 (+/- 0.0)
**Cotton**	72	0.0 (+/- 0.0)	29.6 (+/- 1.2)	3.6 (+/- 0.4)
**Tyvek Micro- Clean Hood**	72	0.0 (+/- 0.0)	18.6 (+/- 0.3)	7.2 (+/- 0.1)
**Tychem QC Suit**	72	0.0 (+/- 0.0)	19.1 (+/- 0.5)	6.6 (+/- 0.1)
**Goggles**	72	0.0 (+/- 0.0)	23.0 (+/- 0.2)	5.5 (+/- 0.1)
**Kimberly-Clark 300 N95 Respirator**	72	0.0 (+/- 0.0)	17.9 (+/- 0.1)	7.0 (+/- 0.0)
**Gloves**	72	0.0 (+/- 0.0)	16.7 (+/- 0.3)	7.3 (+/- 0.1)
**Boots**	72	0.0 (+/- 0.0)	17.3 (+/- 0.1)	7.1 (+/- 0.0)
**Wood**	120	0.0 (+/- 0.0)	20.0 (+/- 0.8)	6.3 (+/- 0.2)
**Plastic**	120	0.0 (+/- 0.0)	18.3 (+/- 0.1)	6.8 (+/- 0.0)
**Steel**	120	0.0 (+/- 0.0)	18.4 (+/- 0.3)	6.8 (+/- 0.1)
**Cotton**	120	0.0 (+/- 0.0)	27.4 (+/- 1.0)	4.2 (+/- 0.3)
**Tyvek Micro- Clean Hood**	120	0.0 (+/- 0.0)	18.3 (+/- 0.1)	6.8 (+/- 0.0)
**Tychem QC Suit**	120	0.0 (+/- 0.0)	18.0 (+/- 0.2)	6.9 (+/- 0.1)
**Goggles**	120	0.0 (+/- 0.0)	24.8 (+/- 0.5)	4.9 (+/- 0.2)

**Kimberly-Clark 300 N95 Respirator**	120	0.0 (+/- 0.0)	17.9 (+/- 0.1)	7.0 (+/- 0.0)
**Gloves**	120	0.0 (+/- 0.0)	18.2 (+/- 1.4)	6.9 (+/- 0.4)
**Boots**	120	0.0 (+/- 0.0)	18.0 (+/- 0.1)	6.9 (+/- 0.0)
**Wood**	168	0.0 (+/- 0.0)	19.5 (+/- 1.0)	6.5 (+/- 0.3)
**Plastic**	168	0.0 (+/- 0.0)	18.9 (+/- 0.1)	6.7 (+/- 0.0)
**Steel**	168	0.0 (+/- 0.0)	18.7 (+/- 0.3)	6.7 (+/- 0.1)
**Cotton**	168	0.0 (+/- 0.0)	27.8 (+/- 0.9)	4.1 (+/- 0.3)
**Tyvek Micro- Clean Hood**	168	0.0 (+/- 0.0)	19.5 (+/- 0.8)	6.5 (+/- 0.2)
**Tychem QC Suit**	168	0.0 (+/- 0.0)	19.1 (+/- 0.1)	6.6 (+/- 0.0)
**Goggles**	168	0.0 (+/- 0.0)	25.3 (+/- 1.0)	4.8 (+/- 0.3)
**Kimberly-Clark 300 N95 Respirator**	168	0.0 (+/- 0.0)	18.2 (+/- 0.3)	6.9 (+/- 0.1)
**Gloves**	168	0.0 (+/- 0.0)	17.4 (+/- 0.4)	7.1 (+/- 0.1)
**Boots**	168	0.0 (+/- 0.0)	18.8 (+/- 0.1)	6.7 (+/- 0.0)

* **VT =** virus titre per mL presented in Log_{10} scale.
† **Ct =** cycle threshold.
‡ **GE =** genome equivalents per mL presented in Log_{10} scale.

Table S2: Ebola virus penetration through select items of PPE measured in infectious virus titre.

Material	VT*
**Inoculation Control**<br>**KC 300 N95 Respirator**	6.8 (+/-0.4)
**Swab Recovery**<br>**KC 300 N95 Respirator**	6.1 (+/-0.1)
**Dry**<br>**KC 300 N95 Respirator**	0.0 (+/-0.0)
**Saturated**<br>**KC 300 N95 Respirator**	0.0 (+/-0.0)
**Mock**<br>**KC 300 N95 Respirator**	0.0 (+/-0.0)
**Inoculation Control**<br>**Tyvek Micro Clean Hood**	6.8 (+/-0.4)
**Swab Recovery**<br>**Tyvek Micro Clean Hood**	6.1 (+/-0.2)
**Dry**<br>**Tyvek Micro Clean Hood**	1.0 (+/-1.5)
**Saturated**<br>**Tyvek Micro Clean Hood**	0.5 (+/-0.7)
**Mock**	0.0 (+/-0.0)

**Tyvek Micro Clean Hood**	
**Inoculation Control Tychem QC Suit**	6.8 (+/-0.4)
**Swab Recovery Tychem QC Suit**	6.6 (+/-0.3)
**Dry Tychem QC Suit**	0.0 (+/-0.0)
**Saturated Tychem QC Suit**	1.1 (+/-1.7)
**Mock Tychem QC Suit**	0.0 (+/-0.0)

* **VT =** virus titre per mL presented in Log_{10} scale.

Table S3: Ebola virus penetration through a surgical mask and N95 respirators measured in infectious virus titre, cycle threshold and genomic equivalents.

Material	VT*	Ct†	GE‡
**Inoculation Control Dry Surgical Mask**	6.2 (+/- 0.5)	14.1 (+/-0.1)	8.9 (+/-0.0)
**Inoculation Control KC 200 N95 Respirator**	5.8 (+/-0.1)	14.6 (+/-0.3)	9.1 (+/-0.0)
**Inoculation Control KC 300 N95 Respirator**	6.2 (+/-0.2)	13.6 (+/-0.3)	9.0 (+/- 0.1)
**Dry Surgical Mask Swab**	0.0 (+/-0.0)	Undetermined	0.0 (+/-0.0)
**Dry KC 200 N95 Respirator Swab**	0.0 (+/-0.0)	Undetermined	0.0 (+/-0.0)
**Dry KC 300 N95 Respirator Swab**	0.0 (+/-0.0)	Undetermined	0.0 (+/-0.0)
**Dry Surgical Mask Face Swab**	0.0 (+/-0.0)	Undetermined	0.0 (+/-0.0)
**Dry KC 200 N95 Respirator Face Swab**	0.0 (+/-0.0)	Undetermined	0.0 (+/-0.0)
**Dry KC 300 N95 Respirator Face Swab**	0.0 (+/-0.0)	Undetermined	0.0 (+/-0.0)
**Saturated Surgical Mask Swab**	0.7 (+/-0.7)	24.3 (+/-1.0)	5.8 (+/-0.3)
**Saturated KC 200 N95 Respirator Swab**	0.7 (+/-0.7)	30.0 (+/-0.2)	4.0 (+/-0.0)
**Saturated KC 300 N95 Respirator Swab**	1.7 (+/-0.9)	23.6 (+/-1.1)	6.0 (+/-0.4)

**Saturated Surgical Mask Face Swab**	0.0 (+/-0.0)	28.0 (+/-2.9)	4.7 (+/-0.9)
**Saturated KC 200 N95 Respirator Face Swab**	0.7 (+/-0.7)	31.6 (+/-2.1)	3.5 (+/-0.6)
**Saturated KC 300 N95 Respirator Face Swab**	2.6 (+/-0.2)	24.2 (+/-0.8)	5.8 (+/-0.3)

* **VT** = virus titre per mL presented in Log_{10} scale.
† **Ct** = cycle threshold.
‡ **GE** = genome equivalents per mL presented in Log_{10} scale.

Table S4: Vesicular Stomatitis-Green Fluorescent Protein Expressing Virus Penetration through Surgical and N95 masks measured in infectious virus particles, cycle threshold and genomic equivalents.

**Material**	**VT***	**Ct†**	**GE‡**
**Inoculation Control Surgical Mask**	6.9 (+/-0.3)	17.3 (+/-0.3)	9.0 (+/-0.2)
**Dry Surgical Mask Swab**	0.0 (+/-0.0)	Undetermined	0.0 (+/-0.0)
**Dry Surgical Mask Face Swab**	0.0 (+/-0.0)	Undetermined	0.0 (+/-0.0)
**Saturated Surgical Mask Swab**	0.6 (+/-0.6)	29.9 (+/-2.2)	3.8 (+/-0.7)
**Saturated Surgical Mask Face Swab**	0.6 (+/-0.6)	32.8 (+/-1.8)	3.0 (+/-0.5)

* **VT** = virus titre per mL presented in Log_{10} scale.
† **Ct** = cycle threshold.
‡ **GE** = genome equivalents per mL presented in Log_{10} scale.

Table S5: Specifications of protective equipment challenged with Ebola virus.

Product Identity	Designation	Protection	Temperature Limit	Humidity Limit	Fit Test Required	Used in Ebola-Makona Outbreak
**Harmony Earloop Face Masks, Adenna 3-ply/ 4 Fold Design**	Surgical Mask	ASTM F 2100-11 Level 1 Barrier BFE>99% PFE>99% @0.1 micron			No	Unknown
**Kimberly-Clark 200 N95 Particulate Filter Respirator and Surgical Mask**	N95 Particulate Filter Respirator and Surgical Mask	ASTM F 1862-120mm Hg ASTM F2101-BFE≥98% ASTM F2299-PFE≥98% @ 0.1 micron 95% filtration efficiency of 0.3 micron particles.	20°C-25°C	< 60% Relative Humidity	No	Unknown
**Kimberly-Clark 300 N95 Fluidshield Particulate Filter Respirator and Surgical Mask**	N95 Particulate Filter Respirator and Surgical Mask	ASTM F 1862: 160 mm Hg ASTM F 2101: BFE≥98% ASTM F 2299: PFE≥98% @ 0.1 micron<br><br>95% filtration efficiency of 0.3 micron particles.	20°C-25°C	< 60% Relative Humidity	No	Yes
**Dupont Tyvek Micro-Clean 2-1-2 Hood and Mask**	Provide light-weight inherent barrier protection against hazardous dry particles and aerosols, and nonhazardous	ASTM F2101		55% Relative Humidity	No	Yes

	light liquid splash					
**Dupont Tychem QC**	Lightweight protection from liquid splashes	ASTM F1670 ASTM F1671	Wide Range	<0.1μg/cm^2 /min->216 μg/cm^2 /min	No	Yes
